# Hemocyte Density Increases with Developmental Stage in an Immune-Challenged Forest Caterpillar

**DOI:** 10.1371/journal.pone.0070978

**Published:** 2013-08-06

**Authors:** Teresa M. Stoepler, Julio C. Castillo, John T. Lill, Ioannis Eleftherianos

**Affiliations:** Department of Biological Sciences, George Washington University, Washington, D.C., United States of America; Oxford University, United Kingdom

## Abstract

The cellular arm of the insect immune response is mediated by the activity of hemocytes. While hemocytes have been well-characterized morphologically and functionally in model insects, few studies have characterized the hemocytes of non-model insects. Further, the role of ontogeny in mediating immune response is not well understood in non-model invertebrate systems. The goals of the current study were to (1) determine the effects of caterpillar size (and age) on hemocyte density in naïve caterpillars and caterpillars challenged with non-pathogenic bacteria, and (2) characterize the hemocyte activity and diversity of cell types present in two forest caterpillars: *Euclea delphinii* and *Lithacodes fasciola* (Limacodidae). We found that although early and late instar (small and large size, respectively) naïve caterpillars had similar constitutive hemocyte densities in both species, late instar *Lithacodes* caterpillars injected with non-pathogenic *E. coli* produced more than a twofold greater density of hemocytes than those in early instars. We also found that both caterpillar species contained plasmatocytes, granulocytes and oenocytoids, all of which are found in other lepidopteran species, but lacked spherulocytes. Granulocytes and plasmatocytes were found to be strongly phagocytic in both species, but granulocytes exhibited a higher phagocytic activity than plasmatocytes. Our results strongly suggest that for at least one measure of immunological response, the production of hemocytes in response to infection, response magnitudes can increase over ontogeny. While the underlying *raison d’ être* for this improvement remains unclear, these findings may be useful in explaining natural patterns of stage-dependent parasitism and pathogen infection.

## Introduction

Insects have developed diverse and efficient defense mechanisms for opposing infections by microbes and parasitic organisms. They possess an innate immune system that is activated upon detection of foreign invaders, leading to the induction of immune responses, which are directed against the replication and spread of pathogens within host tissues [1]. The six main insect immune reactions include (1) epithelial responses that constitute the first line of defense against intruders [2], (2) humoral responses that regulate the secretion of antimicrobial peptides and effector molecules into the insect blood (hemolymph) by the fat body [3], (3) melanization responses that result in synthesis and deposition of melanin around foreign particles following activation of phenoloxidase (PO) [4], (4) hemolymph coagulation responses that seal wounds [5], (5) gut immune responses and gut-associated commensal bacteria involved in the production of reactive oxygen and nitrogen species [6], [7], and (6) cellular immune responses [8]. The latter are modulated by hemocytes (insect blood cells equivalent to mammalian macrophages) that participate in various immune activities, such as cell spreading and aggregation, nodulation, phagocytosis and encapsulation [9], [10].

Insect hemocytes are classified into distinct types based on morphological and functional characteristics [11]. Although the different types of hemocytes are well described in laboratory-reared holometablous insect models including tobacco hornworm (*Manduca sexta*), the common fruit fly (*Drosophila melanogaster*), and various mosquitoes (e.g., *Anopheles gambiae*, *Aedes aegypti* and *Culex* sp.), little is known about the distribution of hemocyte types and their functional properties in insects collected from the wild [12]–[17]. Further, the role of ontogeny in mediating immune responses is not well understood for any non-model invertebrate systems. Differential immune responses over ontogeny may help explain variation in host susceptibility to pathogen infection and parasitism [18]–[20], life-history tradeoffs and costs associated with immune defense [21], [22], and compositional shifts in pathogen and parasite communities over host ontogeny in nature [23], [24].

**Figure 1:**
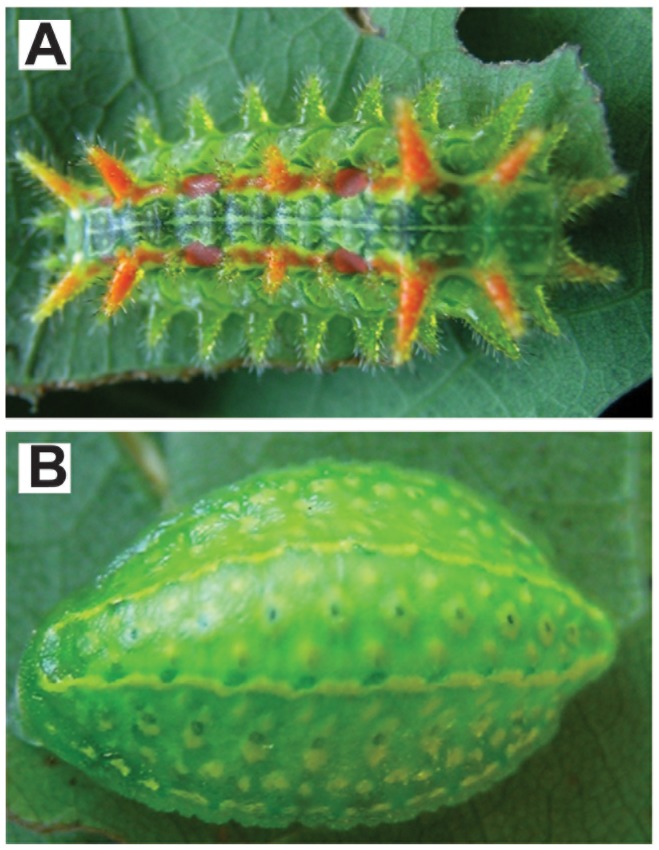
The two caterpillar species. Photo of (A) *Euclea delphinii* and (B) *Lithacodes fasciola* ‘slug’ caterpillars (Limacodidae, Lepidoptera). Photo credit: John Lill.

The “slug” caterpillars *Lithacodes fasciola* Herrich-Schäffer (yellow-shouldered slug) and *Euclea delphinii* Boisduval (spiny oak slug) (Lepidoptera: Limacodidae; *Lithacodes* and *Euclea*, hereafter; Fig. 1) are found in temperate forests of eastern North America in late summer (June–October) [25]. Both *Lithacodes* and *Euclea* caterpillars are highly polyphagous, feeding on smooth-leaved trees of a wide variety of woody plant species [26], and have extremely protracted larval development (i.e., commonly requiring 60–80 days or more from egg hatch to cocoon formation) [27]. In their forest environment, these caterpillars are continuously exposed to natural enemies such as bacteria, viruses, and parasitic wasps and flies (parasitoids) that interact with their immune system throughout their development [28]. As with many larval lepidopterans, parasitoids are a dominant component of the natural enemy community, with incidences of parasitism of 30–50% (and up to 80%) commonly observed in slug caterpillars collected from the field [27]. All of the currently described primary parasitoids of these focal species are restricted to hosts within the family Limacodidae, but attack multiple species within the family [28]. Our previous work in this system has shown that caterpillar size, a proxy for developmental stage, is a primary mechanism by which the two primary parasitoid guilds, wasps and flies, partition their shared host resources. Specifically, parasitoid wasps in the families Eulophidae, Braconidae and Ichneumonidae preferentially parasitize small, early-instar caterpillars, while avoiding and/or unsuccessfully parasitizing large, late-instar slug caterpillars; in contrast, parasitoid flies (Tachinidae) preferentially attack large, late-instar caterpillars [27], [29]. Two general lines of evidence suggest that this consistent ecological pattern may have an immunological basis: (1) small, early-instar caterpillars often have weaker immune defenses against invaders compared to large, late-instar caterpillars [30], [31], and (2) developing wasp parasitoids are much more susceptible to host cellular immune responses than are tachinid fly parasitoids due to important life history differences. For example, compared to wasps, tachinid larvae are not only more mobile, but can highjack the host’s encapsulation response in the construction of a breathing tube, or ‘respiratory funnel’, that provides continuous outside oxygen while larvae develop within the host’s body, thus subverting the oxygen-deprivation caused by melanization responses [32], [33]. The polydnaviruses and venoms often injected into the host by wasp parasitoids are known to alter hemocyte morphology, reduce hemocyte densities, and reduce hemocyte spreading and encapsulation activity, suggesting that wasps are particularly susceptible to host cellular immune responses and have evolved adaptations to mitigate their effects [34].

The aim of the current work was (1) to determine the effect of caterpillar size and age on hemocyte density in naïve and immune-challenged caterpillars of two species of slug caterpillars, *Euclea* and *Lithacodes*, and (2) to characterize the hemocyte morphotypes and their phagocytic properties in both species. We predicted that large, later instar caterpillars would have greater constitutive and post-immune challenge hemocyte density than small, early instar caterpillars.

## Results

### Effect of Caterpillar Size, Age, and Immune Challenge on Hemocyte Density

**Figure 2:**
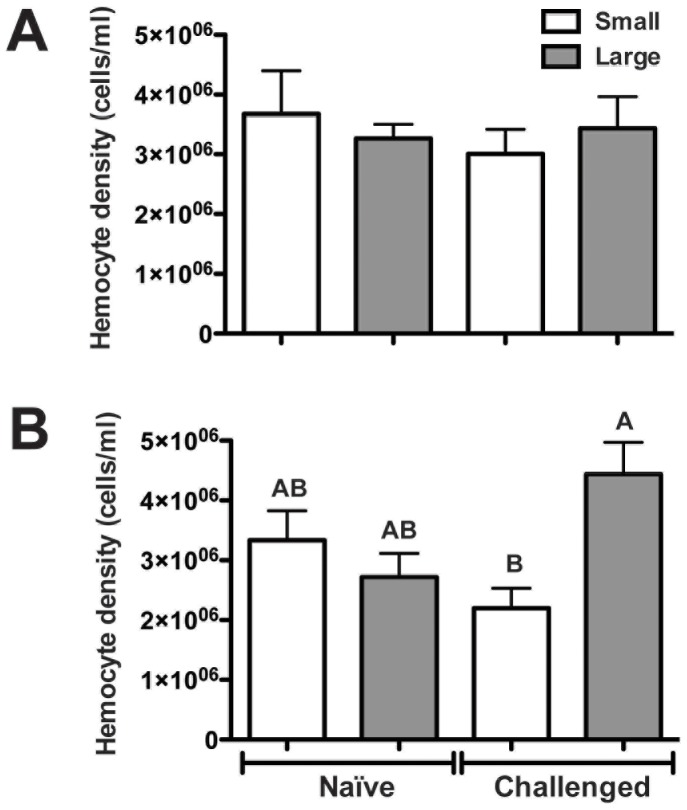
Mean hemocyte density in naïve and immune-challenged slug caterpillars. Hemocyte density (number of hemocytes per ml of hemolymph) from naïve and *E. coli*-challenged small (white bars) and large (grey bars) size caterpillars of (A) *Euclea delphinii* and (B) *Lithacodes fasciola.* Values shown as mean +1 SE, n = 10–28. Different letters above bars in (B) denote significantly different LS means according to Tukey’s HSD test.

Caterpillars were grouped into ‘small’ and ‘large’ size categories based on the typical caterpillar size range of each species from which the most common parasitoid wasps have successfully emerged [27], [29]. The mean body mass ± SE of *Euclea* small and large caterpillars was 71.3±4.4 mg and 253.9±13.1 mg respectively, a highly significant difference (t = 13.20, df = 29.46, *P*<0.0001). The mean body mass ± SE of *Lithacodes* small and large caterpillars was 18.5±2.3 mg and 111.7±5.17 mg respectively, also a highly significant difference (t = 16.46, df = 37.98, *P*<0.0001). There was no effect of caterpillar size category, immune treatment, or their interaction on *Euclea* hemocyte density (hemocytes/ml hemolymph) (F_3,46_ = 0.31, *P* = 0.8162; Fig. 2A). In *Lithacodes*, there was no main effect of caterpillar size (F_1, 76_ = 3.09, *P* = 0.0830), or immune treatment (F_1,76_ = 0.40, *P* = 0.5301) on hemocyte density. However, there was a statistically significant interaction between caterpillar size and immune treatment, as shown by a more than 50% increase in hemocyte density in large vs. small caterpillars in immune-challenged, but not in naïve, *Lithacodes* caterpillars (Interaction: F_1,76_ = 9.52, *P* = 0.0028; Fig. 2B).

**Figure 3:**
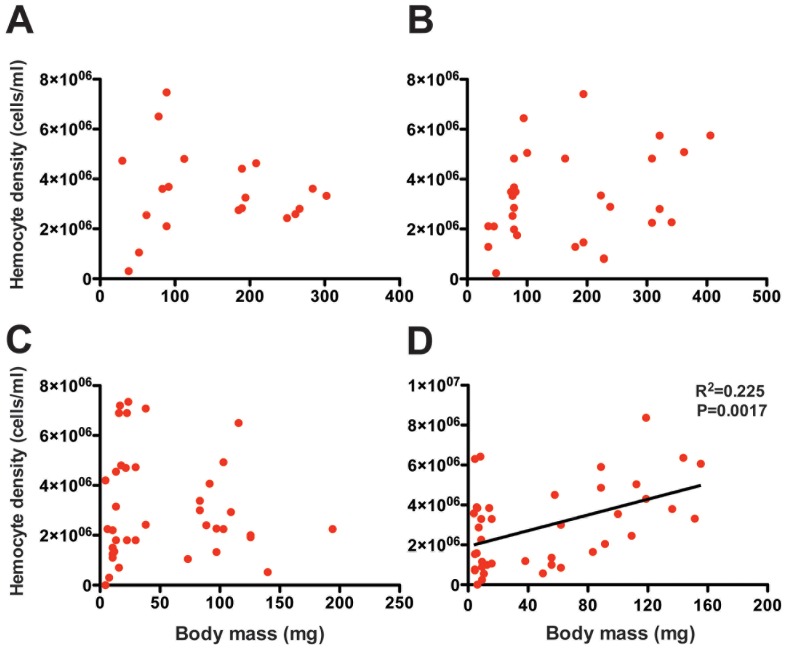
Hemocyte density increases with caterpillar size following bacterial challenge in *Lithacodes*. Regression analysis of caterpillar body mass (mg) and hemocyte density (number of hemocytes per ml of hemolymph) in (A) naïve *Euclea delphinii*, (B) *E. coli*-challenged *Euclea delphinii*, (C) naïve *Lithacodes fasciola*, and (D) *E. coli*-challenged *Lithacodes fasciola* caterpillars. Each data point represents an individual caterpillar.

In *Euclea*, caterpillar mass did not affect hemocyte density in either immune treatment (naïve: r^2^ = 0.00, df = 19, *P* = 0.8055; challenged r^2^ = 0.07, df = 29, *P* = 0.1639; Fig. 3A–B). In *Lithacodes*, caterpillar mass did not affect hemocyte density in the naïve treatment (r^2^ = 0.01, df = 38, *P* = 0.5723; Fig. 3C), but was a positive predictor of hemocyte density in *E. coli*-challenged *Lithacodes* caterpillars (r^2^ = 0.23, df = 40, *P* = 0.0017; Fig. 3D).

Caterpillar age (days since hatching) did not affect hemocyte density in either species or immune treatment (*Euclea* naïve: r^2^ = 0.11, df = 19, *P* = 0.1534; challenged: r^2^ = 0.00, df = 29, *P* = 0.9508; *Lithacodes* naïve: r^2^ = 0.00, df = 38, *P* = 0.6180; challenged: r^2^ = 0.05, df = 40, *P* = 0.1819).

### Hemocyte Morphology

**Figure 4:**
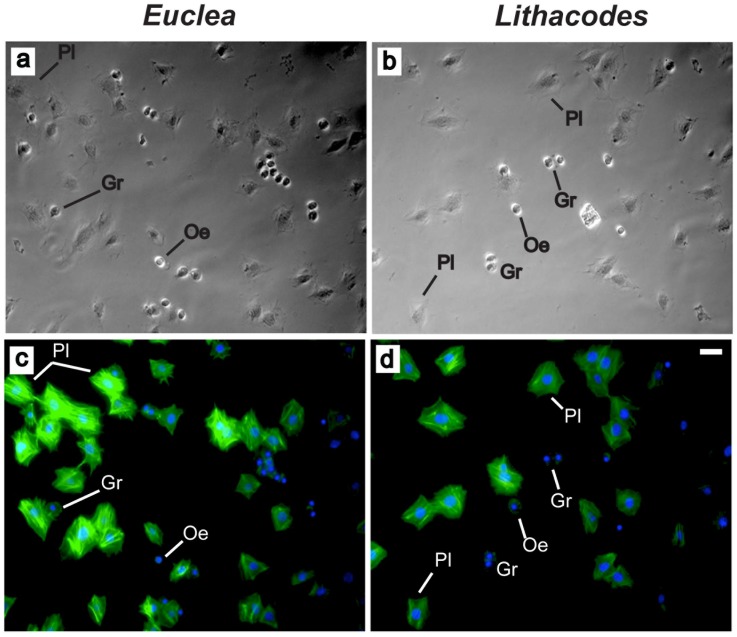
Monolayers prepared from hemocytes extracted from slug caterpillars. Panels (A) and (C), differential interference contrast and fluorescent images of hemocytes extracted from *Euclea delphinii*. Panels (B) and (D), hemocytes extracted from *Lithacodes fasciola*. Hemocytes were fixed and stained with phalloidin (green) cytoskeleton stain and Hoestch 33342 (blue) nuclear stain to reveal cell morphology. Granulocytes (Gr) and plasmatocytes (Pl) displayed spreading and adhesive behavior whereas oenocytoids (Oe) appear to be non-adhesive. Scale bar = 25 µm.

**Figure 5:**
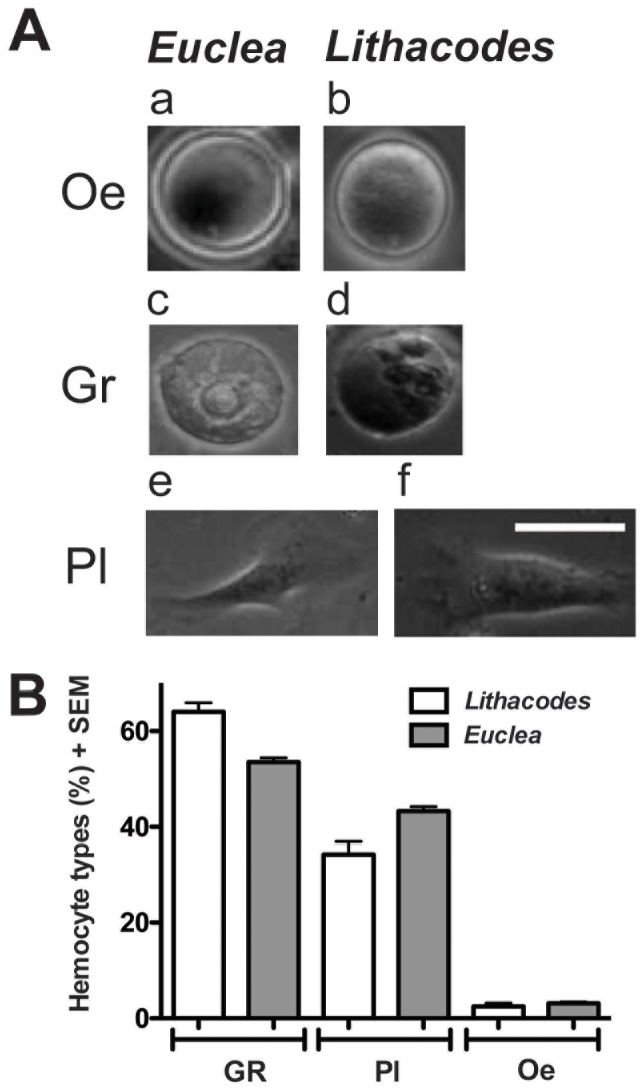
Hemocytes from *Euclea* and *Lithacodes* belong to three distinct morphotypes. (A). Hemocyte types from *Euclea delphinii*: (a) oenocytoids (Oe), (c) granulocytes (Gr), and (e) plasmatocytes (Pl). For comparison, hemocytes extracted from *Lithacodes fasciola* are shown alongside: (b) oenocytoids (Oe), (d) granulocytes (Gr), and (f) plasmatocytes (Pl). (B) Percentages of the different hemocyte types collected from *Euclea* and *Lithacodes* caterpillars, shown as mean ± SE. Cell counts were calculated from six field views per preparation (n = 4 animals per species). A total of 1490 hemocytes from *Euclea* and 961 from *Lithacodes* were counted. Granulocytes were the most abundant followed by plasmatocytes and oenocytoids. Scale bar = 10 µm.

*Euclea* and *Lithacodes* caterpillars both contained three types of hemocytes; granular cells (granulocytes), plasmatocytes and oenocytoids (Fig. 4; panels a, b). Notably, we did not detect the presence of prohemocytes or spherule cells (spherulocytes), which have been found in other lepidopteran species [11]. In both *Euclea* and *Lithacodes*, oenocytoids appeared as small round cells with a smooth surface (Fig. 5A; panels a, b) and a large, eccentrically located nucleus (Fig. 4; panels c, d). Granulocytes were round to ovoid in shape, did not spread extensively like plasmatocytes, and contained granules of variable size and number (Fig. 5A; panels c, d). Plasmatocytes varied in shape, were large in size, were highly adherent, and their plasma membranes often exhibited irregular shapes and formed filopodia and pseudopodia (Fig. 4; panels c, d). Other plasmatocytes were spindle-shaped (Fig. 5A; panels e, f). None of the plasmatocytes contained granules. Ranked by density, in both *Euclea* and *Lithacodes*, the dominant hemocyte type was granulocytes, followed by plasmatocytes, followed by oenocytoids (Fig. 5B).

### Hemocyte Phagocytosis in *Euclea* and *Lithacodes*

**Figure 6:**
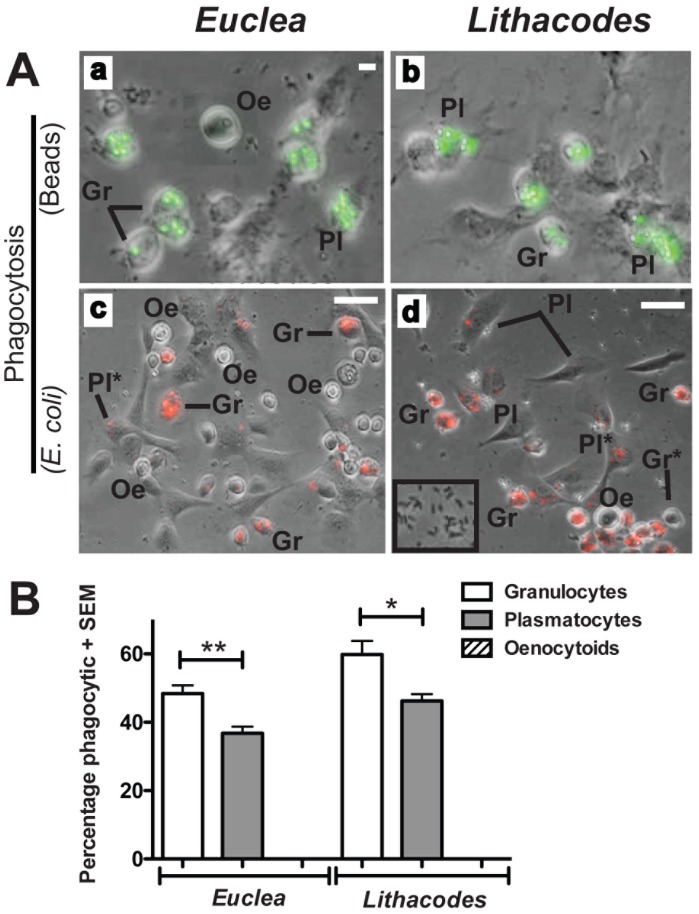
Granulocytes and plasmatocytes from *Euclea* and *Lithacodes* display phagocytic activity. (A) Hemocyte cells from *Euclea delphinii* and *Lithacodes fasciola* are capable of phagocytosing beads (a, b) and *E. coli* bacteria (c, d). Image overlays show hemocytes containing phagocytosed beads (green) in (a) *Euclea* and (b) *Lithacodes* as well as labeled *E. coli* (red) inside phagocytic cells in (c) *Euclea*, and (d) *Lithacodes*. Inset in (d) shows non-fluorescent extracellular bacteria. (B) Percentage of phagocytosis-positive cells for each hemocyte type, shown as mean ± SE. Phagocytosis-positive cells were counted from six different field views per animal (n = 4) for both species. Granulocytes (Gr) were significantly more efficient at engulfing bacteria compared to plasmatocytes (Pl) in both *Euclea* (t = 3.805, df = 6, *P* = 0.0089) and *Lithacodes* (t = 3.093, df = 6, *P* = 0.0213), whereas oenocytoids (Oe) are not phagocytic. ** = *P*<0.01; * = *P*<0.05; Scale bar = 25 µm.

We also used phagocytic activity as an alternative approach for classifying hemocyte types in *Euclea* and *Lithacodes*. In both *in vivo* and *in vitro* phagocytosis assays, only plasmatocytes and granulocytes were phagocytic in both caterpillar species. In the fluorescent bead assay (*in vivo*), we observed fluorescent beads inside of granulocytes and plasmatocytes in both species (Fig. 6A, panels a, b). Similarly, in the *E. coli* assay (*in vitro*), fluorescent *E. coli* bacteria were also only found inside granulocyte and plasmatocyte cells (Fig. 6A, panels c, d). We did not, however, find any fluorescent beads or labeled *E. coli* bacteria inside oenocytoids (Fig. 6A). Notably, quantification of the phagocytosis results showed a significant difference in the phagocytic index of granulocytes and plasmatocytes in both caterpillar species (*Euclea*: t = 3.805, df = 6, *P* = 0.0089; *Lithacodes*: t = 3.093, df = 6, *P* = 0.0213), (Fig. 6B). The percentage of phagocytosis-positive granulocytes was significantly higher than that of plasmatocytes in both species. Hemocyte phagocytosis results combined with cell morphological data strongly suggest the existence of three distinct hemocyte types in *Euclea* and *Lithacodes* caterpillars; oenocytoids (round, non-phagocytic cells), plasmatocytes (cells with a spread cytoskeleton and weaker phagocytic activity), and granulocytes (cells with a less defined cytoskeleton and high phagocytic activity).

## Discussion

Hemocytes play a central role in insect cellular immune responses against foreign invaders, including pathogens and parasitoids. Here we characterized the hemocyte types from two slug caterpillars, *Euclea delphinii* and *Lithacodes fasciola*, and estimated their density at early and late-larval development stages in the presence and absence of infection with a non-pathogenic bacterium.

We found twofold higher hemocyte densities in large, late instar caterpillars compared to small, early instars of immune-challenged *Lithacodes*. This suggests that later instar *Lithacodes* caterpillars may be able to mount a stronger cellular immune response to invaders than caterpillars in early instars. A previous study has also found that hemocyte densities in *Pseudoplusia includens* and *Spodoptera frugiperda* increased with instar [35]. Another study on the innate immune strength across developmental stages of honey bees reported that larvae and pupae had the highest total hemocyte counts, although differences in hemocyte densities among larval instars were not investigated [36]. Our result is consistent with our previous finding that parasitoid wasps, which are highly susceptible to the hemocyte-mediated immune defenses of their hosts, actively avoid or unsuccessfully parasitize large slug caterpillars in nature [27], [29]. In flies in the *Drosophila melanogaster* group, hemocyte densities explained up to 90% of the variation in parasitoid wasp resistance, with higher hemocyte densities linked to greater encapsulation ability of wasp eggs and larvae [22], [37]. However, in our study, caterpillar size did not affect hemocyte density in immune-challenged *Euclea* and the difference in hemocyte density between large and small caterpillars was also greater in *Lithacodes* compared to *Euclea*. Planned future studies using even smaller, earlier instar *Euclea* caterpillars will clarify whether developmental stage and/or species-level differences in response to infection are responsible for this pattern. For example, *Lithacodes* caterpillars may rely on the quick activation and mobilization of their hemocytes whereas *Euclea* caterpillars may initiate other immune mechanisms such as humoral or melanization responses.

The densities of hemocytes in *Euclea* and *Lithacodes* that we report here are in accordance with our previous findings in these two species as well as with values reported in other lepidopteran larvae [38]–[40]. The lack of significant differences in constitutive hemocyte density between small and large naïve caterpillars may suggest that homeostatic mechanisms keep hemocyte densities relatively constant, even as caterpillars grow, but that the ability to mount an induced response improves with size/developmental stage (at least in *Lithacodes*). We did not find an effect of age on hemocyte density, indicating that age is not as good a predictor as developmental stage. Though age and developmental stage are expected to be correlated, differences in diet and genotype can disassociate these two factors, particularly for insects with protracted development.

We used fluorescent hemocyte labeling to characterize the types of hemocytes present, their relative abundance, and their phagocytic ability in both *Euclea* and *Lithacodes* caterpillars. We found that both species contained three distinct types of hemocytes; granulocytes, plasmatocytes and oenocytoids. These main hemocyte morphotypes have previously been recognized and well characterized in other lepidopteran species [11]. Surprisingly, we did not observe spherulocytes which are present in all lepidopteran species studied so far, but have an unknown function, or other minor hemocyte types such as prohemocytes and podocytes [11]. The lack of these hemocyte types in *Euclea* and *Lithacodes* suggests that only three classes of hemocytes, mainly granulocytes and plasmatocytes followed by oenocytoids, are sufficient to perform the cellular immune reactions in these caterpillar species. However, it is currently unknown whether spherulocytes are present in other developmental stages of these caterpillars or whether they are generated under certain environmental or stress conditions. Interestingly, a previous study failed to observe differentiation of spherulocytes from the larval hematopoietic organ of the silkworm, *Bombyx mori* [41]. The authors speculated that spherulocytes may originate from embryonic hemocytes, which is also supported by the finding that spherulocytes exhibit unusual gene expression profiles compared to the other hemocyte types [42]. Limacodidae are fairly primitive within the Lepidoptera, whereas Bombycoidea and Sphingidae (silkworm and hornworms, respectively) are more derived [43]. These differences in phylogenetic position may partially explain the lack of spherulocytes in Limacodidae. Comparative studies examining hemocyte diversity and activity across a broader range of Lepidoptera are necessary to corroborate these early findings.

In both *in vivo* and *in vitro* phagocytosis assays, we consistently found *E. coli* bacteria and beads contained in granulocytes and plasmatocytes but not in oenocytoids in both *Euclea* and *Lithacodes,* suggesting that only granulocytes and plasmatocytes are phagocytic. In general, the primary phagocytic hemocyte type in lepidopteran larvae are thought to be the granulocytes, which are also the first cells to come into contact with invading microorganisms, and secondarily the plasmatocytes that are mainly involved in the formation of capsules or nodules around foreign particles. However, the specific role of plasmatocytes in phagocytosis is not clear and it has been proposed that their ability to perform phagocytosis depends on the experimental conditions [11]. We observed that plasmatocytes from *Euclea* and *Lithacodes* are phagocytic but at lower levels compared to granulocytes. Granulocytes appeared to be the main phagocytic type, followed by plasmatocytes in both species. Our results are in line with previous observations that separate the two cell types into a mostly phagocytic type (granulocytes) and a mostly encapsulating type (plasmatocytes) based on differences in phagocytosis and encapsulation activities [44]. However, it is not uncommon to observe an overlap of cellular functions, as cooperation between granulocytes and plasmatocytes during capsule formation has been observed [45]. Oenocytoids, on the other hand, are responsible for the synthesis and release of the phenoloxidase enzyme upon immune challenge without being phagocytic [39]; as expected, they did not engulf bacteria or beads.

In conclusion, we showed that hemocyte density increases with caterpillar size following immune challenge, with implications for interactions with natural enemies throughout caterpillar ontogeny in nature. Further, we showed that hemocyte types and their function in slug caterpillars differ from those described in lepidopteran model species. While the broader ecological significance of these findings is as yet unclear, the increased hemocyte density in response to immune challenge reported here for late instar *Lithacodes* may partially explain our previously reported pattern of differential resistance of late instar Limacodidae to wasp parasitoids [27], [29]. Future studies will focus on a detailed characterization of encapsulation and nodulation reactions to infection by various pathogenic microorganisms and parasitism by captive wasp colonies in *Euclea* and *Lithacodes* as well as in other wild caterpillars.

## Materials and Methods

### Ethics Statement

*Lithacodes* and *Euclea* caterpillars were reared in the laboratory from colonies originating from moths and caterpillars collected from forests of the greater Washington DC area (Little Bennett Regional Park, MD; Rock Creek Park, Washington, DC; and Patuxent National Wildlife Refuge, MD). Collection of caterpillars was permitted by Wendy Hanley (Park Manager, Montgomery County Parks), Tara Morrison (Park Superintendent, National Park Service), and Sandy Spencer (Supervisory Wildlife Biologist, U.S. Fish and Wildlife Service).

### Caterpillar Rearing

Caterpillars were reared in the lab at room temperature in groups of 5–10 in plastic deli containers (540 ml, Fabri-Kal, Kalamazoo, MI) with a moistened filter paper disc and were fed fresh, excised white oak (*Quercus alba* L.) leaves collected from the field, replaced as needed. While we did not explicitly control for genetic variation, caterpillars of each species used in the experiments were the offspring of at least 10 genetically distinct families, resulting in mixed cohorts.

### Hemocyte Extraction

Live hemocytes were extracted from *Lithacodes* and *Euclea* caterpillars by the methods described in [38]. Briefly, caterpillars were anesthetized on ice, injected with a collection solution [70% Grace’s Insect Media (GM) (Gibco), 10% heat-inactivated Fetal Bovine Serum (FBS) (Hyclone) and 20% Anticoagulant Buffer (98 mM NaOH, 186 mM NaCl, 1.7 mM EDTA, 41 mM citric acid, pH 4.5)], returned to ice for 30 mins, and then “bled” to release hemolymph. The hemolymph-buffer solution (approx. 10–20 µl/caterpillar) was collected, and an equal volume of incubation solution was added (90% GM, 10% FBS) [38]. Collected hemolymph was then used for functional assays and immunostaining experiments described below. The number of hemocytes extracted represents a mixture of circulating hemocytes and any sessile hemocytes that entered circulation as a result of the incubation on ice and changes in cell adhesion caused by the anticoagulant buffer (due to a change in pH).

### Preparation of Bacteria for Immune Challenge

We used a non-pathogenic strain of *E. coli* as an immune elicitor instead of pathogenic bacteria or parasitoid infection to avoid adverse effects conferred by pathogen toxins, virulence factors, venoms or viruses (e.g., Polydnaviruses) on the viability and activity of insect hemocytes [46], [47]. *E. coli* strain K-12 (ATCC# 25254) was grown from a frozen stock on Luria-Bertani broth for 16h at 37°C. Cultures were pelleted for 5 mins at 3,000 r.p.m. and washed 2x using sterile PBS to remove bacterial products (leaving only live cells), and resuspended in PBS. Optical density measures were taken using a Nanodrop® (Thermo Scientific) to determine bacterial concentration.

### Bacterial Immune Challenges

Small and large individuals from both species, *Euclea* and *Lithacodes*, were injected with approximately 1×10^5^ CFU’s of live *E. coli* K-12 strain in PBS using the Nanoject II microinjector (Drummond) and glass capillaries. Animals were incubated as indicated above in rearing cups for 24 hours before hemocyte extraction and hemocyte density estimation.

### Hemocyte Density

To test whether caterpillar size (a proxy for developmental stage or instar) and immune challenge affected total hemocyte density, large and small *Euclea* and *Lithacodes* caterpillars were randomly assigned to one of two treatment groups, control (naïve) and *E. coli-*challenged and their hemocytes were counted. In the control group, hemocytes were extracted from caterpillars as described above, and hemocyte numbers were immediately counted using a Neubauer hemocytometer (Hausser Scientific). The number of hemocytes/ml hemolymph solution (hemocyte density) was calculated from the average of five large hemocytometer squares as in [38]. In the *E. coli*-challenged group, caterpillars were first injected with 1×10^5^ CFUs of *E. coli* strain K12. After *E. coli* injection, caterpillars were returned to their individual plastic rearing containers and allowed to feed on fresh white oak leaves for 24 hours. While these lab-reared caterpillars were not exposed to parasitoids, they may have been exposed to pathogens present on wild-collected leaf material. Hemocytes were then extracted and counted as above.

### Hemocyte Staining for Hemocyte Characterization

To characterize the morphology of hemocyte types present in *Euclea* and *Lithacodes,* hemolymph was extracted from caterpillars (n = 10 *Euclea* and 12 *Lithacodes,* mixed sizes), as indicated above. Hemocytes collected from both caterpillar species were mounted onto slides, fixed, and stained with nuclear and cytoskeleton-specific dyes to reveal cell morphology. Collected hemocytes were incubated for 40 mins in a humid chamber at 30°C on Poly-L teflon coated glass slides (Electron Microscopy Sciences) and fixed with freshly-prepared 4% paraformaldehyde (Electron Microscopy Sciences) in PBS for 20 mins. Each well was washed three times with 1× PBS (pH 7.6) for 5 mins. Cells were then co-incubated with 7 µl of Alexa Fluor 488 Phalloidin Conjugate (1∶200 dilution) (Invitrogen) for staining the cytoskeleton and 5 µl of 500 µg/ml Hoechst 33342 nuclear stain solution (Invitrogen) for 45 mins. Finally, cells were washed twice with 1× PBS and mounted on 50% glycerol in PBS for observation. We determined the different types of hemocytes present in naïve *Euclea* and *Lithacodes* caterpillars by staining the cytoskeleton and nucleus of the extracted hemocytes and using light and fluorescence microscopy to perform morphological analysis of the cells.

### *In vivo* and *in vitro* Phagocytosis Assays

To measure the phagocytic ability of specific hemocyte types in *Euclea* and *Lithacodes* caterpillars, we performed two separate phagocytosis assays; one using fluorescent latex beads (*in vivo*), and a second using fluorescently labeled *E. coli* bacteria (*in vitro*). For the bead assay, caterpillars were injected with a diluted latex bead solution (yellow-green FluoSpheres® Fluorescent Microspheres; bead diameter 2.0 µm, Invitrogen) containing approximately 9.2×10^4^ particles/caterpillar (1∶100 dilution from stock solution) using a glass needle. Caterpillars were then returned to their individual rearing containers and allowed to feed on fresh white oak leaves for 24 hours. After 24 hours, hemolymph was extracted as described above, fixed, slide-mounted, and observed using fluorescent microscopy. Phagocytosed beads within hemocytes were observed and photographed.

For the phagocytosis assay using fluorescent bacteria (live preparation), hemocytes were collected as described above and deposited onto Poly-L Teflon coated glass slides (Electron Microscopy Sciences), and incubated for 45 mins at room temperature to allow the cells to settle. Following incubation, approximately 1×10^5^ pHrodo®-conjugated *E. coli* bioparticles (Invitrogen) were added and the cells were incubated for another 45 mins at room temperature. pHrodo-labeled *E. coli* cells are non-fluorescent outside the hemocytes, but fluoresce bright red once they become phagocytosed. Following the second incubation, live-cell preparations were observed on a fluorescent compound microscope (Zeiss Axioskop; Olympus FVII camera), positive cells (those that internalized bacteria) were counted, and fluorescent and DIC images were taken.

### Data Analysis

We tested for the effect of caterpillar age (days since hatch) and caterpillar body mass (mg) on hemocyte density (hemocytes/ml hemolymph) in the two treatments (naïve or *E. coli-* challenged) using linear regression (each species/treatment combination tested separately). Caterpillars were grouped into ‘small’ and ‘large’ size categories based on the typical caterpillar size range of each species from which the most common parasitoid wasps have successfully emerged [27], [29]. We used two-way ANOVA to test the effects of relative caterpillar size (small or large) and treatment (naïve or challenged; fixed effects) and their interaction on hemocyte density within each species. Tukey’s HSD test was used when means differed significantly. A student’s t-test was used to analyze differences in phagocytic index between hemocyte types. All statistics were performed with JMP® Pro (v.10.0.0).

### Image Analysis

Microscopy images were processed using Adobe*®* Photoshop and Illustrator packages for image compositions.
